# GETTING MORAL ENHANCEMENT RIGHT: THE DESIRABILITY OF MORAL BIOENHANCEMENT

**DOI:** 10.1111/j.1467-8519.2011.01907.x

**Published:** 2011-07-29

**Authors:** Ingmar Persson, Julian Savulescu

**Keywords:** enhancement, moral enhancement, cognitive enhancement, moral bioenhancement, freedom, value of technology, altruism

## Abstract

We respond to a number of objections raised by John Harris in this journal to our argument that we should pursue genetic and other biological means of morally enhancing human beings (moral bioenhancement). We claim that human beings now have at their disposal means of wiping out life on Earth and that traditional methods of moral education are probably insufficient to achieve the moral enhancement required to ensure that this will not happen. Hence, we argue, moral bioenhancement should be sought and applied. We argue that cognitive enhancement and technological progress raise acute problems because it is easier to harm than to benefit. We address objections to this argument. We also respond to objections that moral bioenhancement: (1) interferes with freedom; (2) cannot be made to target immoral dispositions precisely; (3) is redundant, since cognitive enhancement by itself suffices.

## 1. INTRODUCTION

In a recent paper in this journal,^1^ John Harris takes us to task for having ‘fundamentally misunderstood’ the idea of moral enhancement (p. 103). We shall here try to show that he has fundamentally misunderstood our view of moral enhancement and the arguments for it. But before we examine his criticisms, let us summarize what we argued in our paper.^2^

Scientific and technological progress have radically changed the conditions of human life. Through virtually all of their history, humans have lived in societies small enough for everybody to know each other, with simple technology which permitted them to affect only their immediate surroundings, and only in the immediate future. But today, humans live in huge societies with an advanced technology which enables them to affect the environment globally, far into the future, We hypothesized, based on evidence from evolutionary biology and psychology, that the moral psychology of humans is adapted to the former conditions, which have obtained for most of the time the human species has existed. This mismatch is a serious matter because humans now have at their disposal technology so powerful that it could bring about the destruction of the whole planet if misused. Around the middle of last century, a small number of states acquired the power to destroy the world through detonation of nuclear weapons. This century, many more people, perhaps millions, will acquire the power to destroy life on Earth through use of biological weapons, nanotechnology, deployment of artificial intelligence or cyberterrorism. For this reason, the President of the Royal Society, Martin Rees, called his book about the risks posed by the exponentially increasing power of human technology, *Our Final Century*.^3^ We argued that to reduce these risks it is imperative to pursue moral enhancement not merely by traditional means, such as education, but by genetic or other biological means. We will call this *moral bioenhancement*.

## 2. THE RELATIVE EASINESS OF HARMING

We summarized our argument in five points. Harris reviews of all of them, but he expends most energy trying to refute our first point: ‘It is comparatively easy to cause great harm, much easier than to benefit to the same extent.’ He finds this claim ‘totally implausible’ and ‘manifestly absurd’ (p. 106). To get an idea of what motivated us to make this claim, consider an everyday illustration: most readers of this paper probably have access to a car and live in densely populated areas. Whenever you drive, you could easily kill a number of people by ploughing into a crowd. But, we dare say, very few of you have the opportunity every day to save an equal number of lives. Indeed, most of you have probably never had that opportunity, since this kind of situation happens only when, first, a large number of lives is threatened, and, secondly, you are also in a position to eliminate that threat. Harris mentions Mr Schuringa who averted a terrorist attack. But such situations are exceedingly rare. Consequently, it seems indisputable that we are much more frequently in circumstances in which we could kill a number of people than in situations in which we could save an equal number of people. In other words, it is easier for us to kill than to save life.

Harris tries to refute our claim that it is easier to harm than to benefit by citing cases in which someone has saved a great number of lives by averting a serious threat, such as Schuringa's courageous action (pp. 106–7). But it should be clear by now that this is not anything we deny. We are of course not claiming that people are *never* capable of saving as many individuals as would die if a threat were not successfully foiled. We are rather saying that in order to save a number of lives, people have to find themselves in situations in which these lives are under a threat that they could avert, and this is a comparatively rare event, beyond their control. By contrast, they frequently have the opportunity to kill many people. It might be thought that our claim is undermined by the fact that we in affluent countries could save hundreds of lives, cheaply and easily, by donating money to the most cost-effective aid agencies. Most of us could save, relatively easily, 1350 lives^4^ over our lifetime. True, but, again, this is because we happen to find ourselves in special circumstances which are conducive to our providing great benefits, namely a huge global inequality, in which we are vastly better off than many of those who need our help. Also, we could accomplish these greatly beneficial deeds only because there are already in place, due to the work of many good-natured people, highly cost-affective aid agencies. Therefore, we cannot justifiably claim the *whole* credit for the lives saved by our donations.

In their jobs many people, e.g. pilots, doctors, and chefs, have hundreds of lives in their hands. But various institutions, e.g. the law and various security measures, have been implemented to prevent or discourage these people from making destructive use of the opportunities their professions offer. To get a fair comparison to what we could achieve by making donations to welfare agencies, we should imagine that such preventive institutions were absent and that there were instead cost-effective ‘harm’ agencies which offered cheap ways of killing. Imagine how many we could then kill when, as we have seen, most of us could single-handedly and in the everyday run of things kill a two-digit number of people. This is why the moral codes of all human societies around the world include strong proscriptions against killing and harming in-group members, while the injunctions to benefit are fewer and weaker.

Another type of example that Harris provides might seem more compelling than the Schuringa case. He argues that if ‘the preventive measures are permanent’, e.g. measures like vaccines against smallpox, ‘it is surely unlikely that the lives saved will be less than those previously lost in a permanently prevented pandemic’ (p. 10). But here we must first ask whether it is really as easy to vaccinate people against smallpox as it would be to infect them with it (or with a new more lethal strain)?^5^ Smallpox was eradicated by a massive WHO programme which ran from 1967–1977. Note that to be successful, this programme had to cover all countries, even the most undeveloped and mismanaged. During the whole of last century, smallpox killed 300 million people. On a rough, generous estimate, it took 10 years to save 300 million lives. A concerted effort to release (a modified) smallpox virus from Russian or US stores in every major airport would much more quickly result in a global epidemic that would kill at least as many. The fact that it is easier to deliberately spread an infection than to prevent the infection is also shown by the fact that it requires less medical knowledge. English colonial settlers spread smallpox among Australian aborigines and American Indians by the simple means of distributing infected blankets. In contrast, effective protection requires the discovery of a vaccine which takes sophisticated medical research.

However, suppose, for the sake of the argument, that it would be as easy to protect people against smallpox as it is to infect them with it. Then it would follow that it would be as easy to save people from dying of this disease as it would be to kill *by means of this disease*. It still does not follow, however, that it would be as easy to save people from death as it would be to kill them *by some means or other*. For there might be other means of causing death which are more effective and/or accessible^6^ than the smallpox virus, and which are harder to block. For example, ebola virus, an object of biological weapons research, is more deadly than naturally occurring smallpox, being lethal in 90% of cases. The US military has recently provided a vaccine which works in monkeys but it has to be given within 30 minutes of exposure, making it impractical for use in civilian vaccination.^7^ Even if the world were vaccinated against smallpox, everyone would still be susceptible to ebola. If so, it would still be easier to kill than to save life. Another obvious case in point is nuclear weapons. Any half-baked terrorist who had access to weapons-grade plutonium – and there might be enough floating around the former Soviet Union to furnish 20 000 A bombs^8^– could plant dirty bombs in 20 mega-cities (these bombs would be very difficult to detect if encased in lead) and sequentially detonate them. It strikes us as irresponsible to write off our worry about such risks as ‘paranoia’, as does Harris (p. 106).

So far, we have been talking about killing and saving life, but our first claim is about *harming* and *benefiting*. To the extent that their life is worth living, killing people is harming them, and saving their lives is benefiting them. But there are of course other ways of harming than killing and benefiting than saving life: we could harm people by injuring them, depriving them of food and drink, and causing them pain; and we could benefit them by healing their wounds, supplying them with food and drink, and causing them pleasure. In these instances, too, we claim that it is easier to harm than benefit. It should, however, be stressed that simply omitting to harm is not benefiting. If it were, it would not be easier to harm than to benefit, since in general it is very easy not to kill – indeed, easier than to kill. But if omitting to harm were benefiting, then the act-omission doctrine – which states, roughly, that harming is morally worse than omitting to benefit – would be self-contradictory. For it would then imply that harming could be worse than omitting to omit harming, i.e. than harming. Now, our argument does not commit us to the truth of the act-omission doctrine, as Harris seems to think (p. 108), but we do think that benefiting (and harming) should not be so defined that this doctrine becomes self-contradictory. Rather, benefiting (or harming) somebody is acting so that they become better (or worse) off than they would be in the absence of the action. While omissions may be morally equivalent to actions, this does not make omissions the same thing as actions, nor does it turn omission to harm into a benefit.

Let us continue to explore harming by killing and benefiting by saving life. Imagine, contrary to what we have argued, that it would be as easy to save life as to kill; it would still not follow that it would be as easy to benefit as to harm by these means. There are innumerable conditions which are necessary for a person to continue to be alive and, thus, to enjoy the goodness of life. If we remove *any* of those conditions, we kill the person, and thereby deprive her of all the future good that her life would have contained had it not been ended. Thus, by removing any of those conditions, we are guilty of causing her a harm which equals the loss of the goodness of which she is deprived. But if we had instead saved her from death at the same time, we cannot claim credit for all the good that the future has in store for her, since this instance of saving her life is only one of countless conditions which are necessary for her to have a future good life. Consequently, the benefit we would bestow upon someone by saving her life at one single time would be less than the harm we would do her were we to kill her at the same time, for our saving is not sufficient for her to receive the future good life, whereas the killing is sufficient to deprive her of it. Therefore, even if it had been as easy to save life as to kill, which we have argued that it is not, it would not be true that our capacity to benefit would be as great as our capacity to harm by these means.

This is based upon a general point about the conditions for the existence of a complex, organized state, such as human life. There are an indefinite number of conditions which are necessary to keep it functioning. The removal of any of these is enough to disrupt its functioning, but to ensure its function, we have to see to that *all* of these conditions remain in place. Obviously, that is a taller order. The life of a person is like a house of cards. It only takes the removal of one card for the house to fall down, but to keep it standing all or most of the cards must remain in place.

In this argument we have assumed that life is worthwhile, at least better than non-existence. When life is worse than non-existence, the opposite would hold: by killing somebody, we would benefit him much more than we would harm him were we instead to save his life. For this reason, euthanasia could be a benefit. We shall however proceed on the assumption that human life is normally better than non-existence, since we believe that this is the view that most of us would take.

Against this backdrop, consider the fact that a super-power could exterminate all sentient life on this planet by the use of its nuclear weapons. If sentient life is worthwhile, the harm this act would produce by preventing all this value would be colossal, since otherwise worthwhile life might have continued for millenia. In another paper, we describe an act which forever makes it impossible for there to be worthwhile life on this planet as causing *Ultimate Harm*.^9^ No human agent could provide an ultimate benefit, i.e. ensure that there is worthwhile life forever.

Harris contends: ‘if a mad or bad individual can destroy the world instantly by setting off a doomsday machine, then a good consequentialist can save the world as quickly by killing him’ (p. 108). True, but the good consequentialist would not do as much good as the mad or bad individual would do damage, for while the mad or bad individual would make worthwhile life on this planet forever impossible, the good consequentialist would not ensure this. This might require that another mad and bad individual is prevented from implementing his plans tomorrow, or that some natural existential threat be averted, say an asteroid strike, etc. Harris in effect concedes our point when he writes ‘There is no such thing as *ensuring* safety’ (p. 109). Quite right, there are innumerable ways in which things could go wrong, We cannot protect ourselves against all of them. In contrast, there definitely *is* such a thing as ensuring death and the deprivation that it brings along.

## 3. THE VALUE OF SCIENTIFIC PROGRESS AND THE RISK OF ULTIMATE HARM

Harris' objections to the other points of our argument can be dispatched more swiftly. Our third point was: ‘Even if only a tiny fraction of humanity is immoral enough to want to cause large scale harm by weapons of mass destruction in their possession, there are bound to be such people in a huge human population … unless humanity is extensively morally enhanced’ (p. 108). Harris objects that ‘it is not just the wicked that present problems of this sort’ (p. 108). He points out that the ‘incompetence or stupidity’ (p. 108) of those who have access to weapons of mass destruction also presents a problem. We agree, and in our paper we discussed the similar property of negligence (2008: 171–172), but we concentrated on wickedness or malevolence because we wanted to make a case for the need for moral enhancement. However, contrary to what Harris insinuates, our third point does *not* imply that it is *only* immorality or wickedness which presents a problem. Claiming that there is a danger of large-scale harm from weapons of mass destruction unless humanity is morally enhanced (so that immorality is erased or substantially reduced) implies only that this is *necessary* to remove the danger, not that this is sufficient. Our third claim is entirely compatible with other character traits, such as incompetence, negligence, etc. presenting as much of a problem.

Finally, Harris objected to what was our fifth point: ‘Therefore, the progress of science is in one respect for the worse by making likelier the misuse of ever more effective weapons of mass destruction, and this badness is increased if scientific progress is speeded up by cognitive enhancement, until effective means of moral enhancement are found and applied’ (p. 109). Despite the fact that we explicitly talk about worseness *in one respect*, and despite quoting our caveat ‘We have not attempted to settle definitely the balance between … good and bad respects of scientific progress’ (p. 110), Harris takes us to ‘bet against the *overall* utility of scientific advance and cognitive enhancement’ (p. 110; italics added). Thus, in spite of our explicit disclaimers, Harris takes us to assert that scientific progress is for the worse *all things considered*, rather than simply in one respect. Although the single piece of textual evidence he adduces (p. 110) for attributing this more extreme view to us could perhaps be interpreted in this way *in some contexts*, it is uncharitable to do so in a context in which there are other passages, such as the ones quoted above, and others (e.g. 2008: 162), that decisively tell against this interpretation of our position.

In a later paper, however, we do go on to make a stronger claim, though not as strong as the claim that Harris saddles us with. We speculate that at the time, half a century ago or so, when scientific technology provided us with means of causing Ultimate Harm, technological development reached a stage at which it is for the worse *all things considered*, as long as we are not capable of handling those means in a morally responsible way.^10^ Thus, we do not commit ourselves to the extreme claim that technological development *from its inception* (in the Stone Age, say) has been for the worse all things considered.^11^ But not even this stronger claim implies the recommendation that Harris charges us with: that ‘we wait patiently … for the mid to far future perfection of genetic or biological moral enhancement, and … put on hold the cognitive enhancement that might accelerate scientific advance and the discovery and innovation it produces’ (p. 109). Since we regard it as very urgent indeed to discover biological and genetic means of moral enhancement, to remove or reduce the risk of Ultimate Harm that now exists, it would hardly be coherent to recommend that we ‘put on hold the cognitive enhancement that might accelerate scientific advance’ which might be necessary to find the requisite means in time.

The 21st century is a unique century. For the first time, we have means of bringing about Ultimate Harm, and these means are falling into an ever-increasing number of hands. Since there is almost no chance of turning the clock back on our destructive power, the only hope now is to control its deployment, and the chances of that are increased by moral enhancement, which itself is dependent on the continued advance of science.

Harris continues: ‘I don't believe it would be rational to bet on moral enhancement and against our ability to deal with … literally anything, an ability which is likely to stem, with immediate effect, from cognitive enhancement’ (p. 111). But why should we bet on one against the other?^12^ Why can't we have both? Why can't we have scientific research, accelerated by cognitive enhancement, but channel some of it towards finding means of moral bioenhancement?

## 4. THE GROUNDS FOR HARRIS' DISAPPROVAL OF MORAL BIOENHANCEMENT: ITS INTERFERENCE WITH HUMAN FREEDOM, ITS IMPRECISION AND REDUNDANCY

Evidently, Harris has something of an antipathy towards moral bioenhancement, so let us see whether he has any solid grounds for this antipathy. He voices a familiar kind of worry that there is an opposition between our freedom to act and moral bioenhancement. But, unfortunately, he spends less space on articulating this worry philosophically than on literary references to John Milton and William Golding. He does, however, have this to say: ‘The space between knowing the good and doing the good is a region entirely inhabited by freedom … We know how lamentably bad we are at doing what we know we should’ (p. 104), that is, that we are weak willed.

Suppose, first, that our freedom is compatible with it being fully determined whether or not we shall do what we take to be good. Then a judicious use of moral bioenhancement techniques will not reduce our freedom; it will simply make it the case that we are more often, perhaps always, determined to do what we take to be good. We would then act as a morally perfect person now would act.

Suppose, on the other hand, that we are free only because, by nature, we are not fully determined to do what we take to be good. Then moral bioenhancement cannot be fully effective because its effectiveness is limited by our freedom in this indeterministic sense. So, irrespective of whether determinism or indeterminism in the realm of human action is true, moral bioenhancement will not curtail our freedom.

When Harris fears that moral bioenhancement will ‘make the freedom to do immoral things impossible, rather than simply make the doing of them wrong and giving us moral, legal and prudential reasons to refrain’ (p. 105), he seems to think that those who are morally bioenhanced will turn into mindless robots who do not act for reasons. But, in our view, they would rather act for the same reasons as those of us who are most moral today do, and the sense in which it is ‘impossible’ that they do what they regard as immoral will be the same for the morally enhanced as for the garden-variety virtuous person: it is psychologically or motivationally ‘impossible’. People who are morally good and always try to do what they regard as right are not necessarily less free than those who sometimes fail to do so.

Thus, we have to conclude that Harris' fear that moral bioenhancement is at odds with freedom is groundless. But perhaps he has better reasons for his antipathy towards moral bioenhancement. He claims, in connection with combating the immoral attitude of racism, that our ‘resorting to biological or genetic measures … might have unwanted effects’ (p. 105), and he suggests that, for instance, they could ‘weaken kinship ties’ (p. 105). He also writes that he is ‘sceptical that we would ever have available an intervention capable of targeting aversions to the wicked rather than the good’ (p. 105), Harris is, evidently, afraid that we shall throw out the baby with bathwater (p. 105).

In our paper, we identified altruism and sense of justice or fairness as central moral dispositions. It is true that by strengthening altruism and the sense of justice, we would weaken kinship ties to some extent, namely to the extent that they give rise to such things as nepotism, or unjust favouritism of kin – but this effect should not be ‘unwanted’. However, it should not be denied that there are risks associated with the development of techniques of biomedical moral enhancement, but this is also true of such techniques of cognitive enhancement which Harris fervently supports.

A third reason for his antipathy, which could be combined with the second reason, is that means of moral bioenhancement are redundant because, as Harris believes, we can do ‘literally anything’ by means of cognitive enhancement. But, to take a rather simple example, consider paedophilia. By hormonal castration, we can reduce the sex drive of paedophiles and, thus, the risk that they will commit sexual offences against children. There is no form of cognitive enhancement which is as effective. We cannot see that the freedom of paedophiles is thereby reduced, since it reduces their desire to do something that they are not (legally) free to do, namely to have sex with children. But hormonal castration has the drawback of depriving them of sexual enjoyment. Therefore, it would seem desirable that we develop biomedical means which enable us to direct their sexual desires towards adults, so that they could obtain legitimate sexual gratification. It seems unlikely that this could be achieved by cognitive means.

As an example of moral enhancement which could be accomplished by cognitive means, including traditional moral education, Harris mentions racism. He writes that there is ‘good reason to believe that racism can be defeated by such means without resorting to biological or genetic measures’ (p. 105). Now, we do not deny that cognitive enlightenment can be effective against racism. Quite the contrary, we explicitly affirm this, but we add: ‘the mere realization that racism is false is not enough to wash away all xenophobic reactions in our nature’ (2008: 168). As remarked, Harris himself concedes that we are ‘lamentably bad’ at doing what we see as good and right, i.e., we are weak willed, and this is presumably true in the case of racism as well. It is this sort of motivational insufficiency that we are hoping could be addressed by bioenhancement of our moral dispositions. It is unclear how cognitive enhancement could be an effective remedy in this respect, since we are here dealing with people who are assumed to know already what is good and right.

Harris claims that cognitive means of moral enhancement have been so effective that by now ‘racism affects, in a virulent form, only a minority of the world's population’ (p. 105). We are less sanguine. We suspect that anti-racism is not infrequently only a façade covering an underlying xenophobia which under certain circumstances, e.g. when resources become scarce, flares up in excessive violence. For instance, in the 1990s this happened in ex-Yugoslavia and Rwanda. It would be naïve to think it cannot happen again. The fight against racism and xenophobia is surely far from over.

Harris believes that our natural ‘moral endowment’ is ‘extensive’: ‘We have certainly evolved to have a vigorous sense of justice and right, that is, with a virtuous sense of morality’ (p. 103). We do not deny that humans have evolved such a sense, but we think it is largely limited to in-groups as against out-groups. As Frans de Waal puts it: ‘In the course of human evolution, out-group hostility enhanced in-group solidarity to the point that morality emerged … And so, the profound irony is that our noblest achievement – morality – has evolutionary ties to our basest behaviour – warfare’.^13^ This fits in with the ‘shocking statistic that in pre-industrial societies one in three young men is killed in a fight, between men’.^14^ In the pre-industrial Yanomamo tribe which lives in the Amazon, it was found that two out of five men have participated in murder.^15^ Against this horrifying background, the 20th century might appear as a great improvement, in spite of its depressing statistics of violent death. As Stephen Pinker notes, ‘If the wars of the twentieth century had killed the same proportion of the population that die in the wars of a typical tribal society, there would have been two billion deaths, not 100 million.’^16^ However, the positive fact that there are fewer wars and outbreaks of violence in the modern world is counteracted by the alarming fact that weapons become increasingly potent. One outbreak of violence could now be enough to wipe out all life on Earth.

Harris quotes our claim the ‘the core moral dispositions … have a biological basis and, thus, in principle should be within the reach of biomedical and genetic treatment’ (p. 103). To repeat, we take the core moral dispositions to be altruism and a sense of justice. We support the claim that both these dispositions have a biological basis by tracing their evolutionary origin. There is also a striking correlation of occurrence of a sense of justice in identical twins (Persson & Savulescu, 2008: 171) and of altruism (Baron-Cohen, 2003: 114).

It is plausible to think that in general women have a greater capacity for altruism than men. If this psychological difference tracks gender, this is surely good evidence that it is biologically based. It has been argued at length by Baron-Cohen that women as a rule have a greater capacity for empathy than men.^17^ Our conception of empathy is that it is a capacity to imagine vividly what it is like to be another, to think, perceive and feel as they do. On this conception, empathy is merely a component of altruism, as we understand it, since we take altruism to include also a *concern* about how others feel, a concern that they feel good rather than suffer, i.e. a *sympathetic* concern. This is roughly how Baron-Cohen understands empathy^18^ and so his claims about empathy are equivalent to claims about altruism in our terminology.

Baron-Cohen notes that empathy can act as ‘brake on aggression’.^19^ Thus, we should expect that a lesser male capacity for empathy could go with the greater display of male aggression, which is borne out by the statistics of crimes like murder.^20^ Baron-Cohen does not maintain that women are not aggressive at all. His claim is rather that female aggression tends to take the subtler form of backstabbing, social exclusion, etc. instead of direct physical assault, and he observes that this presupposes mindreading.^21^ He also reports that autism, which consists in a deficiency of at least the cognitive aspect of empathy, is ten times more common among men.^22^

If it is right that women are more altruistic than men, it seems that we could make men in general more moral by making them more like women by biomedical methods, or rather, more like the men who are more like women in respect of empathy and aggression. This would not be corrosive of freedom. Such considerations lead us to reject Harris's claim that ‘the sorts of traits or dispositions that seem to lead to wickedness or immorality are also the very same ones required not only for virtue but for any sort of moral life at all’ (p. 104). A low level of empathy and high level of physical aggression are not requisite for a moral life. It is true that a certain amount of aggression might be necessary for moral behaviour: for instance, anger as a response to offences might be necessary to make offenders change their ways. But it should be a degree of anger which is proportionate to the size of the offence and a sense of justice is required to ensure this, as well as ensuring that anger is not directed at people who do not deserve it.

Even if it is right that our moral dispositions have a biological basis, it does not follow that it is impossible to influence them by moral education or enhancing cognition, as Harris implies. However, we have come across a reason for thinking such education and cognitive enhancement cannot be sufficient for moral enhancement: to be morally good involves not just knowing what is good, but being so strongly motivated to do it that this overpowers selfish, nepotistic, xenophobic, etc. biases and impulses.^23^ We hypothesized in our paper that this might be a reason why the degree of moral improvement since the time of Confucius, Buddha and Socrates has been so small in comparison to the degree of technological progress, despite moral education. We need to speed up the pace of moral improvement urgently to prevent the powerful output of technological progress being misused with catastrophic results.

However, we admit that techniques of moral bioenhancement are not imminent. It will take time – quite possibly too much time – to discover and apply such techniques. Our point was simply that it is a matter of such urgency to improve humanity morally to the point that it can responsibly handle the powerful resources of modern technology that we should seek *whatever* means there are to effect this. If, contrary to what we believe, we could achieve this moral improvement by cognitive means, all well and good. But if it takes moral bioenhancement, these should also be sought and applied, alongside the cognitive means. The future of life on Earth might well hinge upon the adoption of this policy.

## Footnotes

1 Moral Enhancement and Freedom, *Bioethics* (forthcoming). Unprefixed page references in the text are to this paper.

2 I. Persson & J. Savulescu. The Perils of Cognitive Enhancement and the Urgent Imperative of Enhance the Moral Character of Humanity. *J Appl Philos* 2008; 25: 162–177. But see also I. Persson & J. Savulescu. 2011 (*Bioethics* 2011; 25: 102–111). Unfit for the Future? Human Nature, Scientific Progress, and the Need for Moral Enhancement, in J. Savulescu, R. ter Meulen & G. Kahane, eds. *Enhancing Human Capabilities*, Oxford: Wiley-Blackwell.

3 M. Rees. 2003. *Our Final Century.* London: Heineman.

4 T. Ord *Giving What We Can*. Available at: http://www.givingwhatwecan.org[Accessed 25 Jan 2011; J. Savulescu. How Many Lives Should I Save? Available at: http://www.practicalethicsnews.com/practicalethics/2010/11/how-many-lives-should-i-save.html[Accessed 25 Jan 2011].

5 In Persson and Savulescu 2008, (*op. cit.* note 2, pp. 167–168) we mention the possibility of modifying smallpox to make it more or less 100% lethal, as has been done with mouse pox.

6 This needs to be made more precise: to most of us most of the time, to some of us some of the time, etc.? But we have to put this lack of precision to the side.

7 http://www.wired.com/dangerroom/2010/06/army-researchers-find-an-ebola-vaccine-but-it-might-onlyvsave-themselves/[Accessed 25 Jan 2011].

8 See R. Posner. 2004. *Catastrophe: Risk and Response*. Oxford: Oxford University Press: 74.

9 I. Persson & J. Savulescu. 2011. The Turn for Ultimate Harm: A Reply to Fenton. *J Med Ethics*. Online First February 22.

10 Ibid.

11 Actually, this extreme claim might not be so implausible as it might seem at first sight. Craig Dilworth writes: ‘it may be suggested that the Upper Palaeolithic (40,000–25,000 BP) constitutes the high point in the human way of life to date … it can fairly be said that we never had it so good before, and we've never had it so good since. Though average longevity was short by modern Western standards, those who survived infanticide and death related to protowar lived to an advanced age, 60 to 70 being quite possible’ (C Dilworth. 2010. *Too Smart for Our Own Good*, Cambridge: Cambridge University Press: 204). Dilworth notes that people at this time, the beginning of human technology, were well nourished, since game was plentiful, and suffered from very few diseases. Consequently, if we compare *average* quality of human life, it may not be higher today, for although many people in modern affluent societies have a higher quality of life than Cro-Magnons had, billions of people in developing countries today lead lives that are of lower quality. What is striking is, then, that the staggering technological advance has done so little to raise the average quality of human life. This supports our claim about the need for moral enhancement, to make a wiser use of this advanced technology. Of course, if we consider the *total amount* of human welfare instead of its average level, it is vastly greater today than in the days of Cro-Magnon, since the human population is now several thousand times bigger. But the huge amount of current human welfare might be bought at the price of less human welfare in the future, since we are exhausting the resources of the planet – unless we are morally enhanced.

12 As one reviewer noted, there are the same reasons to be sceptical about the development of cognitive enhancement.

13 S. Macedo & J. Ober, eds. 2006. *Primates and Philosophers: How Morality Evolved*. Princeton: Princeton University Press: 54–55.

14 S.B. Cohen. 2003: *The Essential Difference: Male and Female Brains and the Truth about Autism*, New York: Basic Books: 35.

15 Ibid:125.

16 http://www.edge.org/3rd_culture/pinker07/pinker07_index.html[Accessed 25 Jan 2011].

17 S. Baron Cohen. 2003. *The Essential Difference: Male and Female Brains and the Truth about Autism*, New York: Basic Books.

18 But only roughly, since he thinks that sympathy is only one example of the affective responses that empathy encompasses (Ibid: 26–27). This is a difference there is not space to sort out here.

19 Ibid: 35.

20 Ibid.

21 Ibid.

22 Ibid.

23 Note, we are not assuming either externalism or internalism about moral judgement. The salient point is that simply knowing what is morally right is not sufficient for doing what is morally right: you also need to be morally motivated. So, in order for people to do what is right, cognitive enhancement is not sufficient, as Harris claims – motivational enhancement is also necessary. In metaethics, there is an extensive debate about whether externalism or internalism is correct, about whether motivation is ‘external’ or ‘internal’ to moral judgement. But both sides admit that weakness of the will (akrasia) is possible (though the fact that the possibility of akrasia is harder to explain on internalism is considered to be a weakness of it). All we require in order to establish that a problem exists is for there to be weakness of the will; and Harris himself admits the existence of weakness of will (p. 104).

## Notes

**Professor Julian Savulescu** holds the Uehiro Chair in Practical Ethics at the University of Oxford. He is Director of the Oxford Uehiro Centre for Practical Ethics within the Faculty of Philosophy. He is Director of the Oxford Centre for Neuroethics, which is one of three strategic centres in biomedical ethics in the UK funded by the Wellcome Trust. He is also Director of the Institute for Science and Ethics, which is one of the ten founding Institutes within the Oxford Martin School at the University of Oxford. In 2009 he was also awarded a major Arts and Humanities Research Council grant on Cognitive Science and Religious Conflict.

**Ingmar Persson** is Professor of Practical Philosophy at Göteborg University, Sweden. His fields of research are ethics and the philosophy of mind and action. His principal publication is *The Retreat of Reason: A Dilemma in the Philosophy of Life* (OUP 2005). With Julian Savulescu he is currently writing a book, *Fit for the Future*, about the mismatch between our moral psychology, which appears to be shaped for life in small communities with simple technology, and the problems we face in modern societies with millions of citizens and a powerful scientific technology.

